# Tailoring DNA Vaccines: Designing Strategies Against HER2-Positive Cancers

**DOI:** 10.3389/fonc.2013.00122

**Published:** 2013-05-14

**Authors:** Cristina Marchini, Cristina Kalogris, Chiara Garulli, Lucia Pietrella, Federico Gabrielli, Claudia Curcio, Elena Quaglino, Federica Cavallo, Augusto Amici

**Affiliations:** ^1^Department of Bioscience and Biotechnology, University of CamerinoCamerino, Macerata, Italy; ^2^Aging Research Centre, G. d’Annunzio UniversityChieti, Italy; ^3^Molecular Biotechnology Center, University of TurinTurin, Italy

**Keywords:** HER2, immunotherapy, DNA vaccines, immunological tolerance, breast cancer

## Abstract

The crucial role of HER2 in epithelial transformation and its selective overexpression on cancer tissues makes it an ideal target for cancer immunotherapies such as passive immunotherapy with Trastuzumab. There are, however, a number of concerns regarding the use of monoclonal antibodies which include resistance, repeated treatments, considerable costs, and side effects that make active immunotherapies against HER2 desirable alternative approaches. The efficacy of anti-HER2 DNA vaccination has been widely demonstrated in transgenic cancer-prone mice, which recapitulate several features of human breast cancers. Nonetheless, the rational design of a cancer vaccine able to trigger a long-lasting immunity, and thus prevent tumor recurrence in patients, would require the understanding of how tolerance and immunosuppression regulate antitumor immune responses and, at the same time, the identification of the most immunogenic portions of the target protein. We herein retrace the findings that led to our most promising DNA vaccines that, by encoding human/rat chimeric forms of HER2, are able to circumvent peripheral tolerance. Preclinical data obtained with these chimeric DNA vaccines have provided the rationale for their use in an ongoing Phase I clinical trial (EudraCT 2011-001104-34).

## Introduction

The ability to drive the immune system’s powerful defense mechanisms to act against cancer is more than just a theory as decades of preclinical studies have demonstrated that the promise of cancer vaccines is being realized. However, while the success of vaccination in preventing infectious diseases is uncontested, the derivation of efficient vaccines against cancer is a more difficult challenge. Although cancer cells express antigens in a way that immunologically distinguishes them from normal cells, most tumors are only weakly immunogenic because most tumor antigens are “self” proteins which are generally tolerated by the host (Finn, 2003). Thus, an effective cancer vaccine must activate the immune system to react against tumor-associated molecules and, in some cases, overcome immunological tolerance to such molecules. Moreover, the increasing instability of transformed cells’ genomes favors the emergence of low immunogenic clones that then no longer express tumor antigens and finally, cancer cells elaborate many defenses against immune attack. They attempt to become invisible to T cells (Lollini and Forni, 2003) by decreasing the major histocompatibility complex (MHC) glycoproteins expression on their cell membrane, and at the same time, suppress immune reactivity via the direct release of transforming growth factor (TGF)-beta, IL-10, and indoleamine 2,3-dioxygenase (IDO), and through the activation of such secretions in myeloid-derived suppressor cells, tumor-associated macrophages, and dendritic cells (DCs). As a consequence, a tumor favors the activation and the expansion of adaptive regulatory T (T_reg_) cells and therefore, the generation of a tolerogenic environment (Cavallo et al., 2011). This ability to evade immune recognition seems to increase as a tumor grows. In fact, the promising results obtained with preventive cancer vaccines in preclinical models are hard to reproduce with advanced cancers when the immune system is already severely weakened. Advances in cancer biology, an increasing knowledge of immune mechanisms and the availability of new animal models that recapitulate several human cancers have all helped to elucidate the critical issues that influence the efficacy of the immune system’s attack on cancer (Finn, 2003; Lollini et al., 2006; Cavallo et al., 2011). Equally important for the rational design of cancer vaccines is the development of new molecular strategies that are able to provide cancer vaccines with the ability to stimulate the immune response against established tumors, besides hampering cancer progression when used at early stages of the disease.

## DNA Vaccines

DNA vaccines offer distinct advantages over other vaccine prototypes. They are stable, relatively inexpensive and simple to purify in large quantities. Recombinant DNA technology allows for the construction of DNA vaccines that encode selected tumor antigens in their native forms or in a modified molecular format, alone, or together with other molecules, to direct and amplify the desired effector pathways. DNA vaccines are simple circles of DNA, principally derived from bacterial plasmids, which contain cDNA coding for the target antigen, a strong viral promoter to drive the antigen expression in mammalian cells and a polyadenylation signal for transcription termination (Liu, 2003). The DNA vaccine is commonly delivered either via the biolistic system (Fynan et al., 1993) or via simple intradermal or i.m. injections which are commonly followed by a short *in vivo* electric pulse (electroporation) to enhance DNA transfection by inducing transient biological membrane permeability (Bodles-Brakhop et al., 2009). Once DNA vaccines enter mammalian cells, antigen synthesis, and presentation occur (Liu, 2003). Professional antigen-presenting cells (APCs), such as DCs, are able to present the transcribed and translated antigen in the proper context of MHC and costimulatory molecules, eliciting both cellular and humoral responses. In addition, bacterial plasmids, unlike mammalian DNA, are rich in unmethylated CpG dinucleotides which activate the innate immune response by the binding with Toll-like receptor 9 expressed on B cells and APCs. Thus, DNA vaccines are effective even when administrated without adjuvants (Krieg, 2002). However, chemokines and cytokines such as granulocyte-macrophage colony-stimulating factor (GM-CSF) are commonly employed to improve protection by DNA vaccines (Nguyen-Hoai et al., 2012).

## Cutting and Sewing up HER2: From Cut-Down to Chimeric DNA Vaccines

Antigen choice is the major determinant of a vaccine’s success. The functional and structural characteristics of the tyrosine-kinase receptor HER2 make it the perfect target for DNA vaccination against cancer. Firstly, HER2 is a transmembrane (TM) receptor that plays a causal role in oncogenic transformations, restricting the emergence of antigen-loss variants. Secondly, HER2 overexpression in several aggressive course carcinomas, unlike its expression in normal tissues, ensures a specific anti-cancer response and minimal risk of autoimmune attack on healthy tissues. Thirdly, HER2 is exposed on the cell membrane and can thus be readily targeted both by antibodies and cell-mediated immunity (Lollini and Forni, 2003). Indeed, vaccines that target HER2, whether they are designed as whole cells, peptides, or DNA expression plasmids, are able to hamper cancer progression when used at early disease stages (Ladjemi et al., 2010). In particular, DNA vaccines directed against HER2 have proven to be successful in the prevention of tumor growth in transplantable tumor models as well as in HER2 transgenic mice (Quaglino et al., 2004, 2005). The induction of effective HER2 antitumor immunity seems to rely on a number of mechanisms which depend on tumor model (Ursini-Siegel et al., 2007). On the other hand, HER2 is a “self” molecule, therefore triggering a stable and strong response to it must circumvent tolerance mechanisms. Several different DNA vaccines have been generated against HER2 thanks to recombinant DNA technology. A rational dissection of the HER2 sequence led to the identification of the most immunogenic portions of the molecule and, eventually, of the crucial epitopes for the induction of immunoprotection. These vaccines had mainly been tested in BALB-neuT mice which are genetically predestined to develop lethal invasive mammary carcinomas with 100% penetrance, high multiplicity (all mammary glands are affected) and relatively short latency (Quaglino et al., 2008). In these mice, the neoplastic transformation of the mammary epithelium is due to the expression of the activated rat HER2 (a mutated form with valine instead of glutamic acid at residue 664 in the TM domain) in the mammary gland.

**Figure 1:**
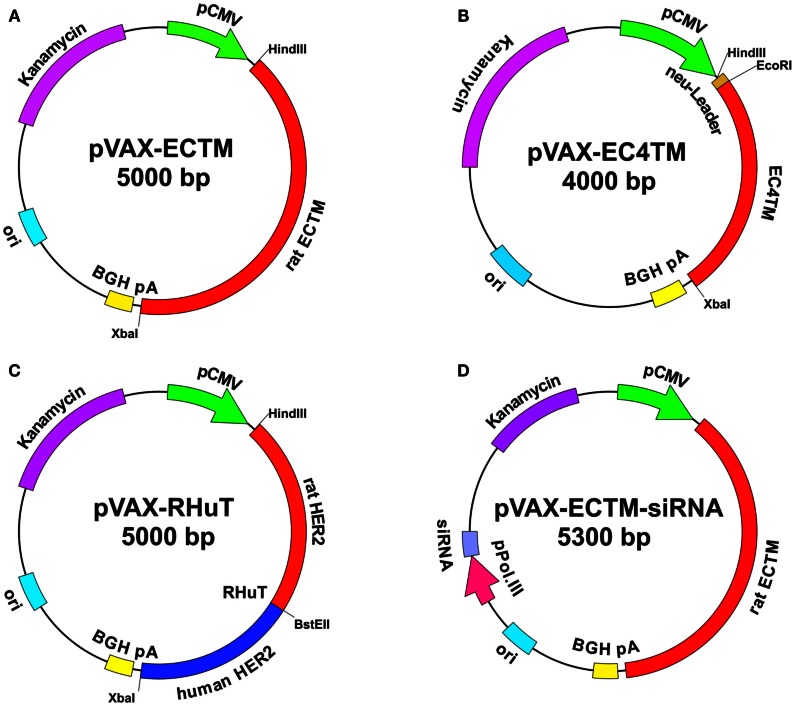
**Maps of anti-HER2 DNA vaccines**. pVAX1 (3.0 kb) (Invitrogen) was used as a backbone. The vectors contain the following elements: human cytomegalovirus immediate-early (CMV) promoter (green) for high-level expression in a wide range of mammalian cells; bovine growth hormone (BGH) polyadenylation signal (yellow) for efficient transcription termination and the polyadenylation of mRNA; kanamycin resistance gene (purple) for selection in *E. coli*; and the origin of bacterial replication (pUC ori, light blue). pVAX-EC-TM **(A)**: the cDNA (about 2 kb) that encodes the extracellular (EC) and transmembrane (TM) domains of rat HER2 was cloned into pVAX1 using *Hin*dIII and *Xba*I restriction enzymes. pVAX-EC4-TM **(B)**: the cDNA that encodes a truncated form of rat HER2, which displays an EC domain that was shortened by 310 NH_2_-terminal residues and a TM domain, was inserted into pVAX1 using *Eco*RI and *Xba*I restriction enzymes, downstream of the leader sequence that had been previously cloned with *Hin*dIII and *Eco*RI. RHuT **(C)**: rat cDNA that encodes the 410 NH_2_-terminal residues of HER2 was cloned into pVAX1 using *Hin*dIII and *Bst*EII restriction enzymes upstream of the human cDNA that encodes the remaining residues of the EC and the TM domains previously cloned using *Bst*EII and *Xba*I restriction enzymes. pVAX-EC-TM-siRNA **(D)**: a silencing module, which generates a short hairpin (sh)RNA under the control of a polymerase III promoter was inserted into pVAX-EC-TM. The interference cassette is composed of the pH1 or pU6 promoter upstream of an insert that specifies a 19–21-nt sequence derived from the target transcript, separated by a short spacer (6–9 nt) from the reverse complement of the same 19–21 nt sequence.

HER2 consists of an extracellular (EC) domain, a single TM-spanning domain and a long cytoplasmic tyrosine-kinase domain. The EC domain is about 630 amino acids long and contains four sub-domains arranged as a tandem repeat of a two-domain unit (Cho et al., 2003). In one of the first studies of HER2-targeted DNA vaccination, it was demonstrated that removing the intracellular domain of this protein and maintaining its TM anchorage improved immunoprotection. In fact, the plasmid that encoded the EC and TM domains of HER2 (Figure 1A) proved to be far superior to plasmids that only encoded the EC domain (secreted form) or the full-length protein (Amici et al., 2000). Most subsequent studies, performed in both wild-type BALB/c mice and cancer-prone BALB-neuT transgenic mice, confirmed the existence of this vaccine’s unique ability to trigger protective immunity toward rat HER2-positive tumors (Rovero et al., 2000; Curcio et al., 2003; Quaglino et al., 2004; Rolla et al., 2008). About 50% of BALB-neuT mice that had been electroporated with the EC-TM plasmid at 10 and 12 weeks of age, when the mammary glands displayed atypical hyperplasia, remained free of autochthonous mammary tumors up to at least 1 year of age, whereas all unvaccinated mice succumbed to mammary cancer within 22–27 weeks (Quaglino et al., 2004).

In order to identify the minimal EC portion of rat HER2 that is still able to elicit protective immunity, sequential deletions of multiples of 240 bp, corresponding to 80 amino acids, were carried out starting from its NH_2_-terminal sequence (Rolla et al., 2008). A first series of DNA vaccination experiments with the resulting seven cut-down plasmids was performed in wild-type BALB/c mice transplanted with syngeneic rat HER2-positive adenocarcinoma cells established from a BALB-neuT mouse mammary tumor (TUBO cells). Significant protection was obtained in mice immunized with the first four cut-down plasmids, while protection declined in mice immunized with shorter fragments. In particular, EC4-TM (Figure 1B), which lacks almost half of the EC domain and exposes only 344 amino acids, protected all vaccinated mice through the induction of anti-rat HER2 antibodies at levels comparable to those in mice vaccinated with the whole EC-TM (Rolla et al., 2008). However, in wild-type BALB/c mice, vaccination triggered a strong immune response because the rat HER2 protein target is a foreign, xenogeneic antigen differing in less than 6% of amino acids from mouse HER2. This immune protection is much more difficult to achieve in cancer-prone BALB-neuT mice, which are tolerant to rat HER2 protein because they express the transgene protein product in their thymus early in life, and for this reason they completely lack T cells that recognize dominant epitopes with high affinity (Rolla et al., 2006, 2010). The progression of the autochthonous rat HER2-positive lesions is accompanied by rampant immunosuppression due to the expansion of T_reg_ cells and immature myeloid cells (Ambrosino et al., 2006). In those mice, only electroporation with EC4-TM hampered BALB-neuT autochthonous mammary carcinogenesis, extended tumor-free survival, and reduced tumor multiplicity, whereas the other cut-down plasmids were completely ineffective. This remarkable protection was not different from that afforded by the EC-TM plasmid and correlated with the induction of anti-rat HER2 antibodies (Rolla et al., 2008). Interestingly, EC4-TM induced a stronger antibody-dependent cell-mediated cytotoxicity (ADCC) response than the whole EC-TM, suggesting that EC4-TM provides accessible critical determinants that may be partially masked in the whole EC-TM (Rolla et al., 2008). Together, these results led to the conclusion that; (i) the first 390 rat HER2 amino acids are those responsible for triggering the protective immunity induced by EC-TM vaccination, (ii) the main mechanism responsible for the antitumor response is represented by anti-rat HER2 antibody production, (iii) the immune protection elicited by DNA vaccines that encode the full-length or truncated HER2 is doomed to decline in tolerant BALB/neuT mice and carcinogenesis is no longer controlled about 3 months after each vaccination course.

Since immunological tolerance against HER2 represents a barrier to effective vaccination against this oncoprotein, the next challenge in the generation of anti-HER2 cancer vaccines is the development of molecular strategies aimed at breaking this tolerance to the HER2 self-antigen.

To further improve the elicited protection, two new chimeric DNA vaccines (RHuT and HuRT) were constructed by combining the two advantages of specificity, ensured by syngeneic portions, and tolerance break, ensured by xenogeneic portions, all in one molecule (Jacob et al., 2010; Quaglino et al., 2010, 2011). In particular, HuRT was derived by cloning the human cDNA fragment that encodes the first 390 NH_2_-terminal residues into the rat EC5-TM cut-down plasmid to regenerate the whole EC domain. Almost symmetrically, RHuT (Figure 1C) encodes a protein in which the 410 NH_2_-terminal residues are from the rat HER2 and the remaining residues from human HER2. Chimeric vaccines displayed superior performance in tolerant BALB-neuT mice (Jacob et al., 2010; Quaglino et al., 2010, 2011). While control mice vaccinated with empty pVAX plasmid developed rat HER2-positive mammary tumors within 27 weeks of age, all mice that had been electroporated at 10 and 12 weeks of age with RHuT or fully rat EC-TM remained tumor-free at 40 weeks. However, 10 weeks later, the protection of mice vaccinated with rat EC-TM decreased to about 50%, while 80% of RHuT vaccinated mice remained completely tumor-free. In both cases, the tumor rejection pattern correlated with high titers of anti-rat HER2 antibodies. In addition, repeated boosting of RHuT vaccination to maintain immunological memory allowed to significantly extend tumor protection (Quaglino et al., 2010). Interestingly, the optimal response was elicited when the NH_2_-terminal portion of the chimeric protein and the corresponding portion on the targeted HER2 ortholog were identical. This quality seems to be an almost absolute requirement whose importance goes beyond the chimeric benefit.

## Clinical Trials

The anti-HER2 DNA vaccines have now entered the clinical phase, although only a few Phase I clinical trials are under way. Among them, one is ongoing to evaluate the side effects and identify the best dose of RHuT delivered using electroporation in patients treated for primary Stage III/IV ErbB-2+ carcinomas of the oral cavity, oropharynx, and hypopharynx (EudraCT number: 2011-001104-34). In this study, whose enrollment is still ongoing, DNA vaccination has been proposed without adjuvants or combined therapies. Two other clinical trials are studying the toxicity and efficiency of a DNA vaccine coding for the HER2 intracellular domain when given together with GM-CSF (ClinicalTrials.gov, NCT00436254) or before immunization with a vaccine made from HER2/neu protein (ClinicalTrials.gov, NCT00363012) in patients with stage III-IV breast cancer or ovarian cancer. However, the first administration of a DNA vaccine encoding HER2 in humans was in a pilot clinical trial of patients with metastatic HER2-expressing breast carcinoma who were also being treated with trastuzumab (Norell et al., 2010). The vaccine used in this study was a pVAX encoding a full-length signaling-deficient version of the oncogene HER2 and was administered together with low doses of GM-CSF and IL-2. This clinical trial demonstrated that the anti-HER2 DNA vaccine was safe, well tolerated after i.m. and intradermal administration and could induce long-lasting cellular and humoral immune responses against HER2 in patients with advanced breast cancer (Norell et al., 2010).

## Hunting Crucial HER2 Epitopes

**Figure 2:**
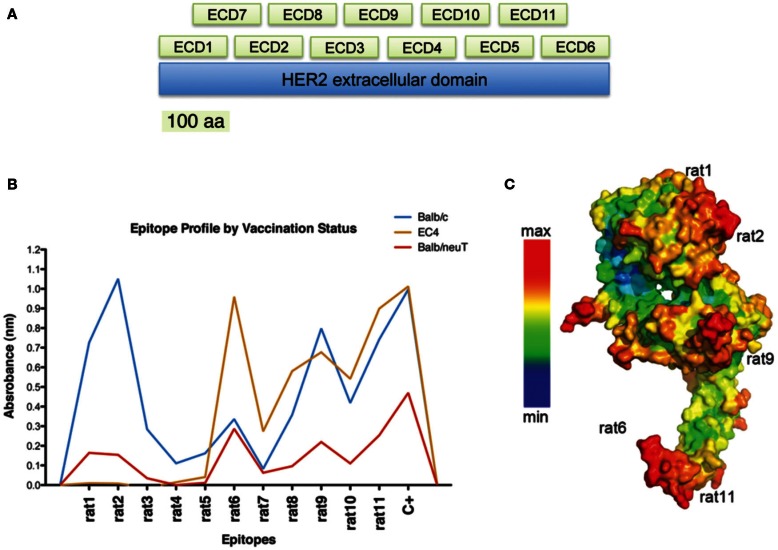
**Identification of relevant conformational epitopes on the HER2 oncoprotein by LFPD**. LFPD structure **(A)**. The EC domain of rat HER2 was divided into 11 fragments of 106 amino acids: 6 contiguous core fragments + 5 fragments that overlap the previous ones. Screening of sera from mice vaccinated with EC-TM plasmid by an ELISA assay based on LFPD **(B)**. Both sera from BALB/c (blue line) and BALB-neuT (red line) mice recognized rat1 (1–106 aa), rat2 (107–216 aa), rat9 (267–376 aa), and rat11 (487–596 aa) conformational epitopes, although tolerant mice sera showed lower reactivity. Sera from mice vaccinated with a truncated form of HER2 (EC4-TM) recognized rat6 (547–656 aa), rat9, and rat11 fragments (orange line). Pepito based analysis of rat HER2 **(C)**. Red zones indicate the maximum probability of finding a conformational epitope. Adapted from Gabrielli et al. (2013).

The generation of an effective vaccine against cancer entails the development and use of new biotechnological tools for the identification of the most immunogenic portions of a target molecule and for the selection of the key epitopes within an oncogenic protein. Considering the fundamental roles of conformational epitopes in effective immunological responses, we engineered a phage-displayed collection of HER2 fragments, that we called Large Fragment Phage Display (LFPD), that reproduces HER2 conformational epitopes (Figure 2A) (Gabrielli et al., 2013). The LFPD was screened using the sera obtained from BALB/c and BALB-neuT mice immunized with the EC-TM plasmid. The screening with sera of BALB/c mice permitted conformational epitopes that correspond to fragments rat1, rat2, rat9, and rat11 to be identified (Figure 2B). This result was confirmed using the sera obtained from BALB-neuT mice, although with a lower reactivity. In addition, sera from mice vaccinated with the EC4-TM truncated form of rat HER2, showed strong reactivity for fragment rat6, as well as for rat9 and rat11 and, as expected, ignored the first part of the molecule (Figure 2B). The strong reactivity toward rat6 may be explained by an immunodominant effect caused by epitopes that might be present in the first part of the molecule (Gabrielli et al., 2013). Remarkably, all the conformational epitopes recognized by the polyclonal antibodies present in the sera of vaccinated mice corresponded to the epitopes predicted by PEPITO software analysis (Figure 2C). In order to selectively induce an immune response against rat2, rat6, rat9, and rat11 HER2 epitopes, we developed DNA vaccines that code for fusion proteins between the murine Fc-region of an IgG2a molecule that acts as molecular support for correct protein folding and each single rat HER2 conformational epitope. Since a TM domain was inserted in each DNA vaccine, the selected antigens were exposed on the cell surface. Preliminary results demonstrate that these epitope-based vaccines are able to induce immune protection against transplanted rat HER2-overexpressing tumors in BALB/c mice, demonstrating that antibodies raised against the selected epitopes can recognize the native rat HER2 protein (Gabrielli et al., 2013). This molecular approach opens the way for the generation of new and potentially more effective B-cell conformational epitope-based vaccines, in which a rational combination of selected xenogeneic and syngeneic epitopes may well lead to an optimization of the immune response.

## Turning Off Network Connections: Implications for Vaccine Design

Dendritic cells play a critical role in immunity and tolerance (Hart, 1997; Steinman and Inaba, 1999). Tumor-induced modulation of DCs function is one of the main causes of tumor immune escape. Tumors produce a range of immunosuppressive molecules which expand or recruit tolerogenic DCs and T_reg_ cells, resulting in the suppression of innate and adaptive immune responses (Jarnicki et al., 2006; von Bergwelt-Baildon et al., 2006). DCs in tumors have limited antigen-presenting function. Inefficient antigen presentation extends to the tumor-draining lymph node and may affect the generation of effective antitumor immune responses (Stoitzner et al., 2008). A new kind of anti-HER2 DNA vaccine has recently been developed as part of a strategy aimed at reverting the immunosuppressive circuits that undermine an effective antitumor immune response. It combines antigen expression with the silencing of immunosuppressive molecules that are responsible for the tolerogenic behavior of DCs (Figure 1D). This double action is associated with two distinct modules; one is the conventional antigen expression cassette, while the other generates specific short hairpin (sh)RNA against an immunosuppressive molecule under the control of a polymerase III promoter (Yen et al., 2009). The interference cassette is usually composed of the pol III promoter (H1 or U6), upstream of an insert that specifies a 19–21 nt sequence derived from the target transcript, separated by a short spacer (6–9 nt) from the reverse complement of the same 19–21 nt sequence (Brummelkamp et al., 2002). The resulting transcript folds back on itself to form a 19 to 21 base pair stem-loop structure. These shRNA structures can be processed by the endogenous endoribonuclease Dicer to generate short interfering (si)RNAs that specify RNA interference process ending with the sequence-specific degradation of messenger RNA (Brummelkamp et al., 2002). RNA interference with the synthesis of negative immune regulators, such as IDO or IL-10, is expected to ensure optimal presentation of the encoded antigen by APCs.

## Conclusion

Cancer vaccines can be designed to prepare the immune system to fight cancer. The choice of the target antigen is the first step to take into account for the generation of a promising cancer vaccine. It is possible to construct multi-task vaccines to improve elicited protection and this is done by combining the most immunogenic portions of a target antigen with molecules that help the immune response, all in one plasmid. However, this technical process is not enough to ensure the success of a vaccine. Cancer vaccines must overcome any immune suppression exerted by the tumor, must break immune tolerance against target antigens and elicit long-term memory, but at the same time they must avoid detrimental autoimmune reactions and as such, the therapeutic schedule, the timing and doses of drug administration are also of crucial importance.

The number of specific strategies we described to generate DNA vaccines against HER2 might be translatable to other vaccines against different target molecules opening the way for the generation of new conceived immunological tools.

## Conflict of Interest Statement

The authors declare that the research was conducted in the absence of any commercial or financial relationships that could be construed as a potential conflict of interest.
